# Exploring the need for a social prescribing pathway in an Australian paediatric allied healthcare intake service: a pilot feasibility and acceptability study

**DOI:** 10.3389/fpubh.2026.1762035

**Published:** 2026-04-01

**Authors:** Lauren Hamill, Anna Kearns, Amy Rogers, Naome Reid, Limin Buchanan, Jahidur Rahman Khan, Natalie Munro, Alison Purcell, Katarina Ostojic, Rachel Walker, Sue Woolfenden

**Affiliations:** 1 Sydney Children’s Hospital, Randwick, NSW, Australia; 2 Sydney Children’s Hospitals Network, Westmead and Randwick, NSW, Australia; 3 Sydney Local Health District, Camperdown, NSW, Australia; 4 University of New South Wales, Sydney, NSW, Australia; 5 Southern Cross University, Lismore, NSW, Australia; 6 The University of Sydney, Sydney, NSW, Australia; 7 Western Sydney University, Campbelltown, NSW, Australia

**Keywords:** access, allied healthcare, centralised referrals, equity, paediatric, social determinants of health, social prescribing, unmet social needs

## Abstract

**Background:**

The social determinants of health (SDH) drive child health inequities. Adverse SDH are experienced at an individual level as unmet social needs. In a paediatric Allied Healthcare system, these unmet social needs are barriers to service access.

**Evidence:**

Social prescribing offers a promising solution. However, this practice is not routinely implemented in Australia.

**Objective:**

To determine whether the Routine Identification of Unmet Social Needs to Unlock Potential (RISE UP) social prescribing model of care is needed, feasible, and acceptable within a paediatric Allied Healthcare intake context.

**Methods:**

A pilot study using mixed-methods approach to design, implement, and evaluate the RISE UP model of care.

**Results:**

Most parent/carers, 114 of 144 (79.2%), agreed to participate in RISE UP, with 74 of 114 (64.9%), reporting one or more unmet social needs. Childcare (*n* = 54, 47.4%) and employment (*n* = 52, 45.6%) were the most common needs. Multivariable analysis showed that each additional year of child age increased the risk of unmet needs by 7% [adjusted relative risk (RR) 1.07, 95% CI 1.03–1.10, *p* < 0.001], and families speaking only non-English at home had a 40% higher risk compared to English-only speakers (adjusted RR 1.40, 95% CI 1.01–1.94, *p* < 0.05). RISE UP was acceptable to parents/carers (97.2%). Staff reported mixed acceptability (55.8%) and feasibility (64.3%).

**Conclusion:**

Unmet social needs identification and referrals pathways in paediatric Allied Healthcare intake services are needed and acceptable to parents/carers. However, further action is required to overcome challenges in integrating these models within existing Allied Healthcare systems in Australia.

## Introduction

The social determinants of health (SDH)—the conditions in which people are born, grow, work, live, and age— drive child health inequities (differential outcomes that are unjust, unnecessary, systematic, and preventable) (1). Child health inequities affect every part of a young person’s health, education, and well-being and these effects last into adulthood (2). Rates of child poverty in Australia have risen sharply post COVID-19 with one in six children affected and the evidence of rising living costs and falling household incomes suggest this number will grow even further (3). For a family, adverse SDH are experienced at an individual level as unmet social needs (e.g., housing instability, food insecurity) (4). In an already overwhelmed paediatric Allied Healthcare system, these unmet social needs are barriers to service access, contributing to the Inverse Care Law–the availability of good medical care varies inversely with the need for it in the population serve (5).

Social prescribing – a person-centred approach that aims to improve health and well-being through connecting people with non-clinical services and supports that address social needs (6) – is expanding in Australia (7, 8) and internationally (9). There is a growing body of evidence demonstrating that sensitive and systematic identification and referral pathways can effectively address unmet social needs (10) and potentially lead to improved health and wellbeing outcomes (11). Over the last decade, health care sector activities related to identifying and addressing patients’ unmet social needs have graduated from being innovative and leading-edge practices to being norms and expectations in the USA (12) and gaining traction in other countries, e.g., UK, Germany, Netherlands, Canada (9). Research indicates healthcare professional generally believe screening and intervening on patients’ socioeconomic risk factors is acceptable and the majority of patients and patient caregivers indicated that social screening is acceptable (13). These findings match our previous work (14), where staff reported high levels of acceptability (93%) and feasibility (98%) and another Australian study (15), where most families (85.7%) reported unmet social needs screening was acceptable. However, this practice is not routinely implemented in Australia or paediatric Allied Healthcare services.

Our team has demonstrated the need, feasibility, and acceptability of a single Allied Healthcare department in a tertiary paediatric hospital in Sydney, Australia, implementing an unmet social needs identification and referral pathway in its intake system, and the potential to scale this across other contexts (14). The current study builds on this work by implementing an unmet social needs identification and referral pathway (i.e., the RISE UP social prescribing model of care) in a multidisciplinary paediatric Allied Healthcare intake line in a non-tertiary community health site in Sydney Local Health District (SLHD)–the Child Health Information Link (16). SLHD is rich in cultural and social diversity and is the home to a large number of culturally and linguistically diverse communities (17). The aim of this study was to determine whether the Routine Identification of Unmet Social Needs to Unlock Potential (RISE UP) social prescribing model of care, an unmet social needs identification tool and referral pathway, is needed, feasible, and acceptable within a paediatric Allied Healthcare intake context in Australia. We based acceptability and feasibility thresholds on research methodologies and consensus-seeking processes (18) where a threshold of 70% or higher indicates agreement on the acceptability, feasibility, or adoption of a claim, and a threshold 75–80% or higher represents robust levels of agreement.

## Methods

### Setting and participants

The Child Health Information Link (CHIL) is the first point of contact, and the single-entry access, for families with children aged 0–12 years who want to access publicly funded paediatric Medical, Nursing, and Allied Healthcare services in SLHD. The CHIL telephone line utilises trained clinicians who provide comprehensive intake and registration to nominated service/s; schedule and book clinic appointments; provide general advice and support regarding childhood development; provide information to referrers and clients regarding alternative services; and assist clients in accessing various services. As such, CHIL staff play an important role in the clients’ intake process and can play a crucial role in tackling health inequalities (19). A referral to one of SLHD’s seven Allied Healthcare disciplines (i.e., speech pathology, occupational therapy, physiotherapy, social work, early childhood social work, psychology, early childhood dietetics), via CHIL, may be the only opportunity that some families have for their unmet social needs to be identified and addressed comprehensively by a health professional.

RISE UP was implemented within the paediatric Allied Healthcare stream of CHIL. CHIL receives approximately 182 paediatric Allied Healthcare phone calls per month. RISE UP was piloted with parents/carers of children (*n* = 114 out of 144) referred to CHIL for paediatric Allied Healthcare services over a 4-month period (17th July-17th November 2024). All CHIL staff (*n* = 9) employed to participate in the Allied Healthcare intake during the study period were also participants.

### Study design

We used a mixed-methods approach to the design, implementation, and evaluation of RISE UP. Accelerating Implementation Methodology (20) was utilised to maximise the chance of successful project implementation. This methodology is a phased implementation framework, used to implement changes based on key principles, that focus on the human elements of change (e.g., staff capacity, sponsorship, communication, cultural fit). Parents/carers were offered the model of care resulting from the Accelerating Implementation Methodology (i.e., RISE UP) as part of the routine telephone intake service. RISE UP was used to identify five unmet social needs (i.e., childcare, employment, housing, food security, and household bills–Supplementary Appendix 1) and unmet needs were addressed via provision of a social care information resource (Supplementary Appendix 2). The social care information resource was developed in consultation with local multicultural service staff to ensure a health literacy and equity lens, specifically with considerations for cultural and linguistic needs. Families were sent the social care information resource regardless of whether they reported unmet social needs to ensure families who did not wish to disclose unmet needs still had access to the resources. At the end of the telephone intake, parents/carers were asked about the acceptability of the RISE UP social prescribing model of care. Following the completion of the data collection period, CHIL staff were offered an anonymous survey to gather information about the acceptability and feasibility of RISE UP.

### Ethics statement

Ethics approval was obtained from the Sydney Children’s Hospitals Network Human Research Ethics Committee (2024/ETH00421). Two methods of consent were used for parents/carers: a waiver of consent for identification of unmet social needs and verbal consent for the acceptability survey. An implied consent approach was applied for CHIL staff participation in the delivery of the model of care and the online acceptability and feasibility survey at the completion of the data collection period.

### Data collection

#### Demographic details

Demographic data were sourced via parent/carer reports. Child age was calculated by subtracting their date of birth from the date of the intake phone call. Sex of child was categorised as male and female based on the reported information. The variable for language spoken at home was grouped into three categories: “English only,” “Mixed” (both English and non-English), and “Non-English only.” Area-level socioeconomic status (SES) was determined using residential postcode of an individual and derived from the Australian Bureau of Statistics’ Socio-Economic Indexes for Areas (SEIFA) Index of Relative Socio-Economic Disadvantage (IRSD) (21). The IRSD reflects social and economic conditions of people and households within an area, focusing on relative disadvantage, where low scores indicate relatively greater disadvantage. In this study, residential area SES were divided into quintiles: Most disadvantaged, Disadvantaged, Middle, Advantaged, and Most advantaged.

#### Unmet social needs

The unmet social needs screening tool used in our formative work (14), adapted from the WE CARE tool (22), was further refined to suit the local context and implemented as the RISE UP social prescribing model of care (Supplementary Appendix 1). Five key domains of unmet need were assessed: childcare, employment, housing, food, and utility bills. Each domain included variables categorised into three response groups: “yes (need),” “no,” and “other (potentially indicate need)”. Data were collected and stored using Research Electronic Data Capture (REDCap), a secure web-based platform for online survey and database management. A copy of the responses was saved in each child’s electronic medical record in the SLHD.

#### Parent/carer acceptability

Parent/carer acceptability was assessed immediately following the RISE UP screening using a single question: “Is it ok for health staff to ask families questions about unmet basic needs?.” Variables were categorised into three response groups: “yes,” “no,” and “not sure.” Parents who responded, “yes,” were asked an additional question regarding modes of delivery (i.e., phone, in person, online form). Responses were recorded in REDCap.

#### Staff acceptability and feasibility

Staff acceptability and feasibility were measured using the Acceptability of Intervention Measure and the Feasibility of Intervention Measure (23). Four items were used to measure acceptability and four items were used to measure feasibility (Table 1). *Each measure has five response options, ranging from ‘completely disagree’ to ‘completely agree’. Higher scores indicate greater acceptability and feasibility. Scales can be created for each measure by averaging responses. No items need to be reverse coded.* Both tools are validated and reliable measures of implementation outcomes and can be adapted for different populations, contexts, and interventions (24). The anonymous 8-item online survey was distributed via email with a REDCap link.

**Table 1:** Staff acceptability and feasibility survey and responses (*N* = 9).

Survey questions		Individual staff member responses									% of staff who completely agree or agree
		1	2	3	4	5	6	7	8	9	
Acceptability Intervention Measure (AIM)	SDH screening meets my approval.										67%
	SDH screening is appealing to me.										56%
	I like SDH screening.										33%
	I welcome SDH screening.										67%
											Average = 55.8%
Feasibility Intervention Measure (FIM)	SDH screening seems implementable.										67%
	SDH screening seems possible.										67%
	SDH screening seems doable.										67%
	SDH screening seems easy to use.										56%
											Average = 64.3%

Key: Completely disagree or disagree = red; Neither agree nor disagree = orange; Agree or completely agree = green.

### Statistical analysis

The primary outcome of interest was unmet need. To simplify analysis and enhance interpretability, responses indicating “yes (need)” and “other (potentially indicate need)” were combined into a single category coded as “1,” while “no” responses were coded as “0.” This binary classification distinguished individuals with identified needs from those without. Following this, we added these five variables with two labels and aggregated to create an unmet need score ranging from 0 to 5, where 0 represents no unmet needs, and 5 indicates unmet needs across all domains. This unmet need score was further grouped into two classes: 0 (no unmet needs) and 1 (at least one unmet need) to identify patterns and associations between unmet needs and other variables in our study. Descriptive statistics were calculated for variables considered in this study, including frequency, percentage, mean, and standard deviation. The distribution of unmet need scores across different sociodemographic groups (i.e., child sex, child age, language spoken at home, residential area SES) was analysed. Associations between sociodemographic factors and unmet needs were estimated using a modified Poisson regression model within a generalised estimating equation framework, accounting for clustering at the residential area level to produce robust and reliable estimates. A multivariable model was fitted, incorporating all variables together, and adjusted relative risk (RR) with 95% confidence interval (CI) were reported. All analyses were conducted using R (25).

## Results

During the data collection period 728 families contacted CHIL and 144 were offered RISE UP (19.7%). Of those offered RISE UP, 114 of 144 families (79.2%) agreed to participate. Sociodemographic data was not recorded for 7 records as these referrals were for families, not individual children, therefore, sociodemographic analysis was based on a sample size of 107 (Table 2). The sample had a higher proportion of male children (*n* = 66, 61.7%) compared to female children (*n* = 41, 38.3%). The average age of the child participants was approximately 3.6 years with a SD of 2.8 years. Most families (*n* = 88, 82.2%) reported speaking only English at home, while 8 families (7.5%) spoke a mix of English and non-English and 11 families (10.3%) spoke only a non-English language. The SES distribution indicated that 15.9% of families were from the most disadvantaged areas, and 34.6% were from the most advantaged areas.

**Table 2:** Distribution of sociodemographic features of this sample (*N* = 107).

Sociodemographic factors	Count (%)
Child sex	
Female	41 (38.3%)
Male	66 (61.7%)
Age (year)	
Average (SD)	3.6 (2.8)
Language spoken at home	
English only	88 (82.2%)
Mixed (English and non-English)	8 (7.5%)
Non-English only	11 (10.3%)
Area SES	
Most disadvantaged	17 (15.9%)
Disadvantaged	6 (5.6%)
Middle	22 (20.6%)
Advantaged	25 (23.4%)
Most advantaged	37 (34.6%)

SD, Standard deviation; SES, Socioeconomic status.

**Figure 1:**
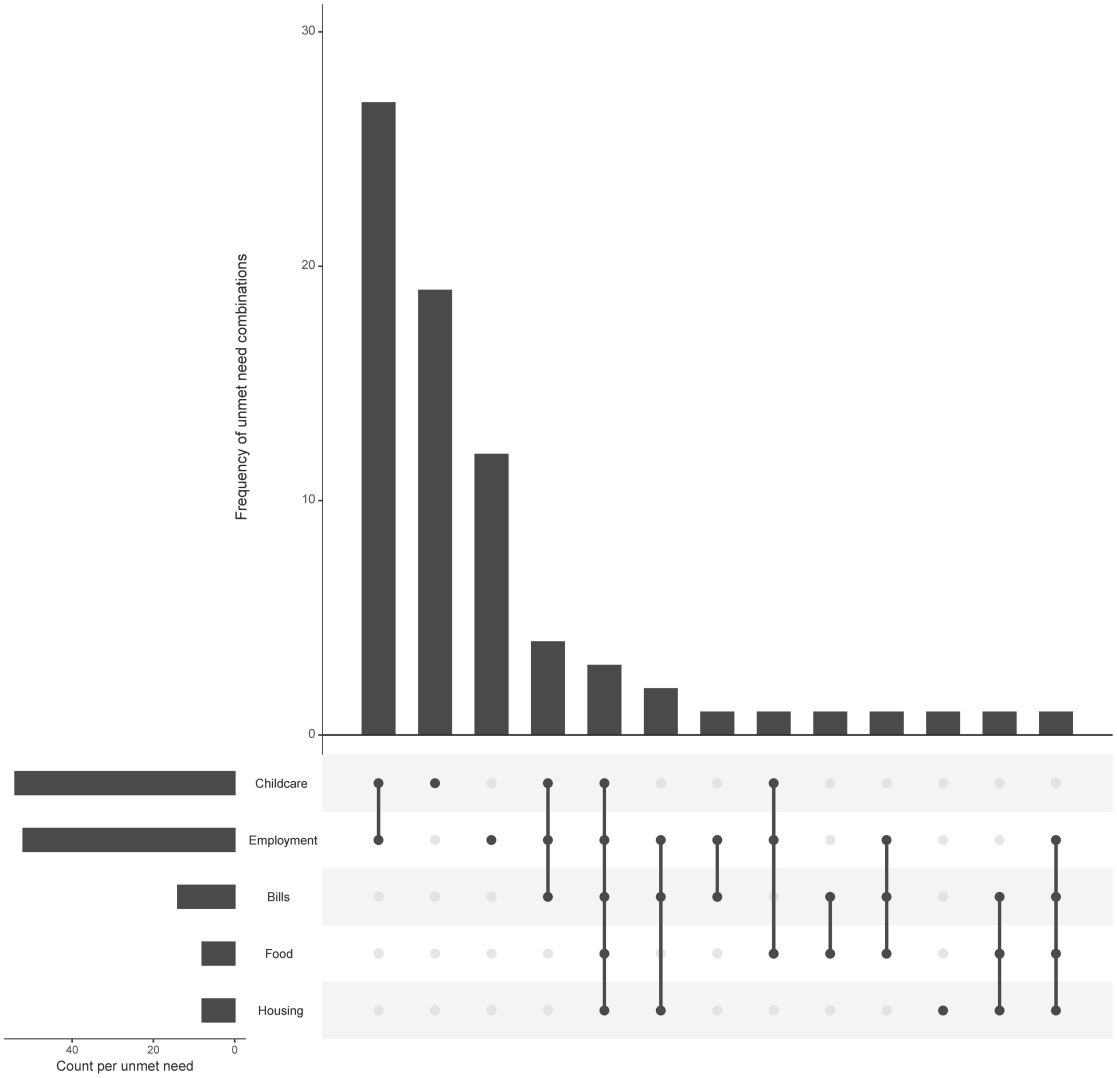
Frequency of unmet need combinations.

A significant portion of families (*n* = 40, 35.1%) reported no unmet needs. However, most families (*n* = 74, 64.9%), reported between 1 and 5 unmet social needs out of a possible 5 areas of needs (Figure 1). Specifically, 32 families reported one unmet need, 29 reported two, 9 reported three, 1 reported four, and 3 reported all five needs. The most commonly reported unmet need was childcare (*n* = 54, 47.4%), followed by employment (*n* = 52, 45.6%), bills (*n* = 14, 12.3%), food (*n* = 8, 7.0%), and housing (*n* = 8, 7.0%). Notably, 27 families (23.7%) reported unmet needs in both childcare and employment. On 28 occasions, families responded by saying, “maybe” when asked about a particular social need. As previously mentioned, these responses were coded as “other” and combined with “yes” responses. The domain-wise distribution of “other” responses was as follows: childcare (5.3%), employment (7.0%), housing (1.8%), food (4.4%), and bills (6.1%). All families reporting unmet needs related to food or bills also reported at least one additional unmet need.

Multivariable regression analysis identified significant associations between unmet needs and two sociodemographic factors: child age and language spoken at home (Table 3). Each additional year of child age was associated with a 7% increase in the risk of unmet needs (adjusted RR 1.07, 95% CI 1.03–1.10, *p* < 0.001). Families speaking only a non-English language at home had a 40% higher risk of unmet needs compared to those speaking only English (adjusted RR 1.40, 95% CI 1.01–1.94, *p* < 0.05). Child sex and residential area SES were not statistically significantly associated with unmet needs.

**Table 3:** Sociodemographic factors associated with unmet need (no vs. any unmet need) using modified Poisson regression model.

Sociodemographic factors	Adjusted RR	95% CI	*p*-value
Child sex			
Female (Reference)	1.00		
Male	0.93	0.75–1.16	0.5399
Child age (years)	1.07	1.03–1.10	<0.001
Language spoken at home			
English only (Reference)	1.00		
Non-English only	1.40	1.01–1.94	<0.05
Mixed (English and non-English)	1.21	0.75–1.96	0.4312
Area SES			
Most advantaged (Reference)	1.00		
Most disadvantaged	0.93	0.76–1.13	0.4524
Disadvantaged	0.79	0.47–1.33	0.3845
Middle	0.83	0.63–1.09	0.1782
Advantaged	0.94	0.69–1.29	0.7058

RR, Relative risk; CI, Confidence interval; SES, Socioeconomic status.

*Parent/carer acceptability:* Of the 114 families who agreed to participate in RISE UP, 109 families elected to answer a follow up question regarding parent/carer acceptability. Parents/carers (*n* = 106 of 109) reported high levels of acceptability (97.2%) of the RISE UP social prescribing model of care. Although RISE UP was implemented via phone, parents/carers (*n* = 106) who found RISE UP acceptable, indicated various modes of delivery were also acceptable (i.e., phone = 100%, in person = 96%, online form = 86%).

*Staff acceptability and feasibility:* All CHIL staff (*n* = 9) eligible to participate, completed the survey. Overall, 55.8% of CHIL staff reported RISE UP as acceptable and 64.3% reported it as feasible (Table 1).

## Discussion

This study demonstrates that unmet social needs identification and referral pathways in paediatric Allied Healthcare intake contexts in Australia are needed. Most families in this study reported unmet needs in one or more of the five domains (childcare, employment, housing, food, and bills). High rates of unmet needs relating to childcare and employment indicated potential challenges in balancing work and family responsibilities. The burden of unmet needs across multiple domains highlights the importance of comprehensive support systems that address various aspects of well-being in order to better support health service access, and child health and development.

Findings indicated a significantly increased relative risk of unmet social needs for families who speak only non-English at home. These results are consistent with international literature (26) that link limited English proficiency with increased rates of social needs. Likely reasons include living in a dominant English-speaking country where policy, systems, and services are primarily delivered in English, which contributes to linguistic and cultural barriers that may restrict access and utilisation of health, social care, and community resources. To address this issue, race should be viewed as a social and political construct that can serve as a proxy for social disadvantage (27) and anti-racism principles must be applied to social care research (28) to ensure the interventions, developed to address inequities, do not unintentionally create further disadvantage.

Another finding indicated that for each additional year in a child’s age, the relative risk of unmet needs significantly increased. There are multiple potential causal pathways for this result. Families of older children may present with more unmet social needs as these concerns are potential drivers for delayed presentation to clinical services. Alternatively, this may be due to the increased financial burden of older children on families. This result highlights a service delivery triage and prioritisation complexity – where there is need to support older children who may have additional unmet needs while also adhering to guidelines and policies that typically focus on prioritising early intervention of younger children (29). In the development of a service delivery prioritisation matrix, multiple factors including age at time of referral, clinical complexity, priority population status, and number and type of unmet needs must all be integrated.

Area SES was not statistically significantly associated with unmet needs. This result contrasts with literature (30) that reports low SES status is linked to a higher prevalence of social needs. In our study, 57.9% of families were from high (i.e., advantaged/most advantaged) SES areas and 21.5% of families were from low (i.e., most disadvantaged/disadvantaged) SES areas. This is interesting considering the clear association between SES and developmental vulnerability in children (31). Either families from lower SES were not asked the RISE UP questions in this study or children from lower SES areas are not being referred to paediatric Allied Healthcare services. Future clinical redesign work will need to address this issue and ensure paediatric Allied Healthcare services are meeting the needs of the local population.

Compared with our previous work (14), families in this study reported higher rates of unmet social needs (i.e., 64.9% vs. 11%). However, compared to another study focusing on paediatric populations with developmental disabilities (15), rates of unmet needs (i.e., 64.8%) were similar. A potential reason for the higher rates of unmet social needs relates to the local contexts in which each study was set, and the structural and systemic factors that have shaped each environment (32). In communities known to have high prevalence of social needs, research suggests clinicians may wish to consider strategies that do not rely on screening and disclosure (33). In our current study, we sent the social care information resource to families regardless of whether they reported unmet social needs to ensure families who did not wish to disclose unmet needs still had access to the resources. Research indicates 49% of clients share social care information with family, friends, and other members of their community (34)–a mechanism that could potentially amplify the effects of the intervention.

RISE UP was highly acceptable to families. Positive parent/carer acceptability ratings in this study (i.e., 97%) were consistent with international literature that has found patients and patient caregivers generally find social screening to be acceptable, though relatively little research has explored the perspectives of racially, ethnically, and linguistically diverse patient populations (13).

Compared to our previous research, staff reported lower rates of acceptability (i.e., 55.8% vs. 93%) and feasibility (i.e., 64.3% vs. 98%). Possible explanations were discussed with CHIL staff as part of the Accelerating Implementation Methodology and were found to match barriers documented in the literature (35). Barriers to implementation included the climate (i.e., timing of the study, increased and competing demands, staff shortages), systems (i.e., workflow and information technology processes), and cultural fit (i.e., motivation)–highlighting the need for further workforce training, capacity building and co-design. It is possible these factors contributed to the discrepancy between the approximate number of phone call referrals made during the data collection period (i.e., 728) and the number of families who were offered RISE UP (i.e., 144). This highlights a Reach gap–a difference between the number of families offered the model of care and those eligible (36). This gap poses a problem as the results may not be generalisable to the entire target population (37) and inequitable procedures could inadvertently undermine the intended promotion of health equity (38–40). Considering almost half of the SLHD population speak a language other than English a home (17) but only 17.8% of the study sample were recorded as speaking a mix of English and non-English (*n* = 8, 7.5%) or only non-English (*n* = 11, 10.3%), further work is required.

### Strengths

Several strengths of this study were identified. These included: (a) a focus on localised iterative improvement activities that aim to identify and address unmet social needs to support service access, strengthen integrated care, improve patient care and outcomes, and reduce inequities; (b) an understanding of the cultural and linguistic diversity within the dataset, and recognition of race as a social and political construct that can serve as a proxy for social disadvantage (41); and (c) stratifying by residential area SES–we recognise the relationship between place, racism, and early childhood development, and acknowledge the structural and systemic factors that shape environments and thus influence child development (32).

### Limitations

Limitations of the study need to be noted. The limitations include: (a) the relatively small sample size of parents/carers and staff; (b) the low Reach and Representativeness of the sample; (c) evaluation of the attitudes and perceptions of parents/carers about RISE UP immediately after asking the questions, by the staff who asked the RISE UP questions; (d) a lack of outcome data measuring any benefits RISE UP may have on families’ unmet social needs; (e) limited tailoring/translation of the social care resource pack for different population partners who have been subject to structural racism; and (f) acknowledgement that addressing individual-level social needs fails to address the upstream wealth and power inequities underlying those needs (4).

### Implications for practice

This study demonstrates that unmet social needs identification and referral pathways in paediatric Allied Healthcare intake contexts in Australia are needed and are acceptable to parents. Using the RISE UP as a social prescribing tool allowed for person-centred unmet social needs to be identified and a care pathway to be provided. Despite parents/carers finding being asked the RISE UP questions at intake acceptable, many of the staff asking the questions did not find using the tool acceptable or feasible. Further work is needed to reduce the barriers to implementation which may in turn increase acceptability and feasibility for staff to use RISE UP. Perhaps, the Reach gap, viewed through a behavioural economics lens (42) may hold promise for future directions to improve evidence based practice (43) through examining the cognitive processes (i.e., attention, memory, effort, perception, intrinsic motivation, and extrinsic motivation) that facilitate or impede action. In addition, for staff to have an increased awareness of families’ social conditions in the health care sector that may influence health and health care utilisation outcomes (12) it is imperative that health staff have a systematic process for identifying and addressing unmet social needs, adequate workforce training and capacity building opportunities, cross-sectoral policy, and authentic partnerships with community members and organisations.

### Implications for future research

Further development of RISE UP, with parent/carer input, is essential and psychometric assessment of the tool would also be valuable. Following continued quality improvement and implementation, future research investigating the impact of RISE UP including the Reach and Effectiveness for families, impact on service utilisation, and economic implications for families and the health service would be beneficial.

## Conclusion

This study highlights unmet social needs identification and referrals pathways in paediatric Allied Healthcare intake services are needed and acceptable to parents/carers. However, further action is required to overcome challenges in integrating these models within existing paediatric Allied Healthcare systems in Australia.

## Data Availability

The datasets for this article are not publicly available due to concerns regarding participant/patient anonymity. Requests to access the datasets should be directed to the corresponding author.
