# Supplementary material for: Exploring the need for a social prescribing pathway in an Australian paediatric allied healthcare intake service: a pilot feasibility and acceptability study
Source: Front Public Health. 2026 Apr 1;14:1762035. doi: 10.3389/fpubh.2026.1762035 (PMC13081777; doi:10.3389/fpubh.2026.1762035)
Supplement: Supplementary file 1 [file Data_sheet_1.pdf]

<u>RISE UP SDH Screening Tool</u>

"As the last part of our call today, I have 6 quick questions about everyday needs that we're asking <u>all</u> families. We're asking these questions because we know children do well when parents/carers have access to these everyday needs."

Basic need	Question	Response		
		Yes	No	Other
Child care	**1. Do you need child care for your child/ren?**			
Paid Work	**2. Do you currently need paid work/more paid work?**			
Housing	**3. Are you homeless or worried that you might be in the future?**			
Food	**4. Do you have enough money to buy the food you need for your family?**			
Household Bills	**5. Do you have enough money to pay your household bills (e.g., electricity, water)?**			
Government benefits & vouchers	**6. Do you want information about government benefits and vouchers?**			

"Thank you for answering those questions. At the end of this phone call, as part of our service I will <u>email/send</u> you an information sheet about community resources to help with everyday needs. Hope this information is helpful."

NOTE: All CHIL staff members must be orientated to SLHD escalation pathways for highly urgent social needs (e.g., homelessness, food security) and child protection matters. Escalation pathways include, but are not limited to, immediate consultation with the CHIL Manager and Child Wellbeing Unit.

Study Name: RISE UP
Document: RISE UP SDH Screening Tool
Ethics Number: 2024/ETH00421
Version 2: April 2024
