# Supplementary material for: Exploring the need for a social prescribing pathway in an Australian paediatric allied healthcare intake service: a pilot feasibility and acceptability study
Source: Front Public Health. 2026 Apr 1;14:1762035. doi: 10.3389/fpubh.2026.1762035 (PMC13081777; doi:10.3389/fpubh.2026.1762035)
Supplement: Supplementary file 2 [file Data_sheet_2.pdf]

INFORMATION AND FREE SERVICES TO SUPPORT YOU AND YOUR FAMILY WITH...

We hope this information is helpful

INTERPRETER/TRANSLATION SUPPORT

Please call 131 450 if you need an interpreter. Ask for an interpreter in your preferred language and then they will connect you to the service.

Available 24 hours, 7 days a week.

'ASK IZZY' WEBSITE

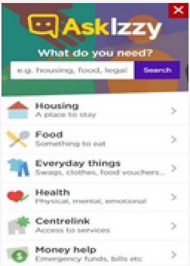

A free website to help you find local services to help with housing, food and more. Available across Australia.

If you are with Telstra and Vodafone, you can access the website even if you don't have phone data, credit or Wi-Fi.

*Available in English only.

www.askizzy.org.au

INFORMATION ABOUT CHILD CARE

Starting Blocks - find information about where child care is available in your local area, the costs, and what services they provide (for example, meals are included at childcare).
www.startingblocks.gov.au/

Child Care Subsidy - you may be able to get help with the cost of childcare
www.servicesaustralia.gov.au/child-care-subsidy

Start Strong Program - you may be able to pay less fees for your child's preschool (before they enter primary school). Check if you are eligible.
education.nsw.gov.au/early-childhood-education/operating-an-early-childhood-education-service/grants-and-funded-programs/start-strong-funding/start-strong-for-families

Study Name: Routine Identification of Social Environments to Unlock Potential (RISE UP) via the Child Health Information Link (CHIL): Is it needed, feasible and acceptable?
Document: RISE UP community resources handout
Ethics: 2024/ETH00421
Version 2: April 2024

GOVERNMENT VOUCHERS & BENEFITS

Information in your language -
Read, listen to or watch information in your language about Australian Government payments and services.
www.servicesaustralia.gov.au/information-your-language?context=64107

NSW Government “Savings Finder” -
There are vouchers and ways you can lower the cost for transport, education, electricity and water bills from the New South Wales Government.
www.service.nsw.gov.au/campaign/savings-finder

Parenting Payment -
You might be able to get income support as a parent or carer of a child. Check if you are eligible.
www.servicesaustralia.gov.au/parenting-payment

Single Income Family Supplement -
A yearly payment of up to $300 might be available, if only one person is earning money in your family.
Check if you are eligible.
www.servicesaustralia.gov.au/single-income-family-supplement

‘FAMILY CONNECT AND SUPPORT’

Free service connecting families to local services to help with housing, food and more. Individual information and advice.

Available across NSW.

Call: 1800 066 757 (8:30am-5:30pm)
Email: familyconnectandsupport@barnardos.org.au
Website: www.barnardos.org.au

FREE EMERGENCY TELEPHONE SERVICES

Link2home Homelessness - a 24-hour information and referral telephone service for people who need emergency housing
- 1800 018 444

Domestic Violence Line - a 24-hour crisis counselling and referral service for women, including trans women, who are experiencing domestic, family or sexual abuse
- 1800 656 463

Lifeline 24-hour telephone counselling and mental health support service
- 13 11 14

Mental Health Line - a 24-hour telephone service for anyone in New South Wales to get mental health advice and connect to mental health services near you
- 1800 011 511
